# Modified Marshall Score: An Underutilised Prognostication Tool for Acute Pancreatitis

**DOI:** 10.7759/cureus.96842

**Published:** 2025-11-14

**Authors:** Charles Her Yi Ling, Richard Bond, Simon East, Richard Young

**Affiliations:** 1 General Surgery, York and Scarborough Teaching Hospitals NHS Foundation Trust, Scarborough, GBR

**Keywords:** acute pancreatitis, glasgow-imrie score, modified marshall score, organ dysfunction, organ failure, risk stratification

## Abstract

Introductions

Acute pancreatitis (AP) remains an increasing cause of mortality in the United Kingdom (UK) with a multitude of aetiologies. Nevertheless, it is known that organ failure due to systemic injury occurs as a complication in the early stage of 20% of AP cases. Early detection and proper risk stratification are paramount for clinicians to make informed decisions. The modified Marshall (MM) score is the default risk stratification tool to assess organ dysfunction in acute pancreatitis patients in local practice.

Aims/Methods

This review retrospectively evaluated the utilisation of the Modified Marshall score amongst acute pancreatitis patients admitted under the General Surgery department of a local district hospital over a period of two years. Our primary goal was to determine the proportion of patients with a completed Modified Marshall score during admission clerking. For our secondary goals, we then risk-stratified each AP case and compared it with the actual patient outcome. Electronic records, discharge letters, and surgical handover sheets were thoroughly assessed to facilitate the data collection process. A questionnaire, which further explored the reasons for underutilisation, was sent to all clerking clinicians and practitioners.

Results

A total of 127 acute pancreatitis patients were admitted with a mean age of 57.1±16.6 years, a 1.4:1 male-to-female ratio, and a median serum amylase of 377±18. Out of 127 cases, only 24 (18.9%) patients were risk-stratified using the Modified Marshall score. We received 19/21 survey responses. Common reasons for underutilisation were unfamiliarity with scoring components, utilisation complexity involving unit conversion, work demand, and preference over other scoring systems. To address the issues, we simplified the MM score formula and designed new unit conversion guidance for the fractional inspired oxygen (FiO₂) component. Following the implementation of our new measure, another review measured an improvement in MM score utilisation over a month in 7/10 patients.

From our retrospective risk stratification, the proportion of patients being risk stratified as severe pancreatitis was 6.30%, 9.45% and 0.79%, respectively, via the Modified Marshall score, Glasgow-Imrie score, and CT severity index. Amongst those scoring low Modified Marshall score <2 (119 patients), 95.8% (114) patients did not progress to requiring intensive therapy unit (ITU) admission or emergency exploratory surgery or drainage.

Conclusion

The modified Marshall score is currently underutilised in our local clinical practice. Although scoring systems are deemed less superior than the evaluation of experienced clinicians, the MM score remains a reliable scoring system with high negative predictive value (NPV) to help surgical trainees to objectively risk stratify AP cases and facilitate more appropriate ITU referrals.

## Introduction

Acute pancreatitis (AP) is defined as inflammation of the exocrine pancreas leading to acinar injury, which subsequently harbours both local and systemic inflammatory responses [1]. To date, acute pancreatitis still remains a common but significantly deadly disease with a mortality rate of 7%, which is expected to increase to 20% if local necrosis is diagnosed and potentially up to 60% if persistent organ failure is present [1-3].

Organ failure due to systemic injury is often expected to occur in the early stage of 20% of AP cases within 72 hours after initial presentation [4]. This can manifest as failure of an organ system, including respiratory, cardiovascular, or renal, and exacerbation of preexisting disorders such as COPD or heart failure [5]. However, organ failure signs in the setting of AP remain under-recognised or treated but with significant delays due to challenging early detection and proper risk stratification despite multiple well-established scoring systems available [6].

The modified Marshall score is both a widely recognised severity score included in the Revised Atlanta Classification and a recommended risk stratification tool for our local clinical practice [5,7]. It objectively quantifies the risk of organ failure in three major systems, i.e., cardiovascular, respiratory, and renal, via basic clinical parameters such as simple renal function tests, blood pressure, and oxygen requirements [7]. A score of ≥2 in any organ system is highly predictive of severe organ failure and warrants the need for proper escalation of care before alarming overt organ failure develops. The primary goal of the review was to determine the proportion of acute pancreatitis admissions with a completed Modified Marshall (MM) score during admission clerking. Secondary objectives were to (1) retrospectively risk-stratify each AP patient using multiple different risk stratification scores such as the MM score, Glasgow-Imrie score, and CT Severity Index, and (2) compare our findings with the actual patient outcome [7-9].

## Materials and methods

This review retrospectively evaluated the utilisation of the Modified Marshall score amongst acute pancreatitis patients admitted under the General Surgery department of a local district hospital over a period of two years (2023 to 2024). Electronic records, discharge letters, and surgical handover sheets were thoroughly assessed to facilitate the data collection process. Data extraction was performed by three independent reviewers. Discrepancies were resolved by consensus. Missing data were excluded from relevant analyses. Retrospective scores (Glasgow-Imrie, CT Severity Index) were calculated based on available documentation, and incomplete parameters were treated as missing data.

We included only patients who are above 18 years old admitted with a primary diagnosis of acute pancreatitis, defined by at least two of the following: characteristic abdominal pain, serum amylase/lipase >3x upper limit of normal, or radiological evidence of acute pancreatitis. Exclusion criteria encompassed patients below 18 years old, chronic pancreatitis flares where baseline organ dysfunction pre-existed, patient transfers in >48 hours after initial presentation, and cases with insufficient data.

Additionally, a questionnaire was also designed and distributed amongst all clerking clinicians for the General Surgery department to further explore the reasons for underutilisation of the MM score. The questionnaire contains multiple-choice and free-text questions, exploring participants’ grade, awareness, or familiarity of the MM score, frequency of MM score utilisation, and possible reasons for MM score underutilisation (Figure 3, Appendix). The responses were collected over a month period.

## Results

Within our review period, we found that 127 patients were admitted with acute pancreatitis under general surgery directly from the emergency department. Our patients were of a mean age of 57.1±16.6 years, with a 1.4:1 male-to-female ratio and a median serum amylase of 377±18 (Table 1). Two-fifths of our patients, 56 (44.1%), suffered two or more active comorbidities during the admission. Out of 127 patients, only 24 (18.9%) were risk-stratified with the Modified Marshall score.

**Table 1:** Patient characteristics at baseline. Data are expressed in median ± standard deviation. The sum of patients from each specific individual comorbidity exceeds the total number of patients with active comorbidities, as some patients presented with multiple active conditions. CKD: Chronic kidney disease, SCr: Serum Creatinine, SBP: Systolic Blood Pressure

Parameter	Value
Total Number of Patients, n(%)	127 (100)
Age, y	57.1±16.6
Sex (male), n(%)	74 (58.2)
Patient with ≥2 Active Comorbidities, n(%)	56 (44.1)
Aetiologies of Acute Pancreatitis, n(%):	
Biliary	64 (50.3)
Alcohol-Related	28 (22.0)
Others: Drugs, ERCP, Hypertriglycerides, Idiopathic	35 (27.6)
Active Comorbidities, n(%):	
Cardiovascular Adverse Events	21 (16.5)
Electrolyte Imbalance	12 (9.4)
Metabolic Disorders	9 (7.1)
Infection of Multiple Origins	8 (6.3)
Others	18 (14.2)
Baseline CKD≥3, n(%)	24 (18.9)
Serum Amylase, (iU/L)	377 ± 18
SCr, (umol/L)	78 ± 52
SBP, (mmHg)	127 ± 27

**Figure 1:**
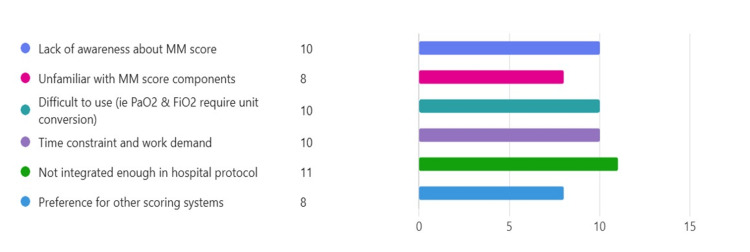
Survey outcome for reasons of underutilisation of the Modified Marshall Score. Common reasons given by surgical clinicians were lack of awareness, lack of familiarity with score components, utilisation complexity, and lack of integration in hospital protocol.

A total of 21 clinicians were surveyed in a one-month period, comprising surgical care practitioners, foundation doctors, core surgical trainees, and surgical registrars, with 19 responses received. Prominent reasons identified for the lack of utilisation of the MM score during surgical clerking were a lack of awareness and unfamiliarity with the components of the MM score. It was commonly agreed in the respondent group that this problem could potentially be due to the lack of integration within the local hospital guidelines (Figure 1). Interestingly, another common reason identified was the complexity of the MM score, and that risk stratification in time-constrained settings can be challenging. A component of the MM score that young clinicians often struggled with was the oxygen requirement calculation, which required manual unit conversion due to unit differences.

From our retrospective risk stratification, the main proportion of our patients, 119 (93.7%), scored an MM score of less than two and were risk stratified as low risk of progression to organ failure. Using different risk stratification models, the proportion of patients being risk stratified as severe pancreatitis was 6.30%, 9.45% and 0.79% via the Modified Marshall score, Glasgow-Imrie score, and CT severity index, respectively. Amongst those scoring low on the modified Marshall score <2 (119 patients), 95.8% (114) of patients did not progress to requiring ITU admission, emergency exploratory surgery, or endoscopic drainage (Table 2).

**Table 2:** Compared the risk-stratified grades using the Modified Marshall score with the actual patient outcome. ITU: Intensive therapy unit, n: Number of patients, %: Percentage of patients

Row Labels	Patients, n (%)	
MM<2:		119 (93.7)
No Progression	104 (81.9)	
Progression- Did not Require ITU Admission	10 (7.9)	
Progression- Required ITU Admission	4 (3.1)	
Progression- Required ITU Admission & Emergency Exploratory Surgery	1 (0.8)	
MM>2:		8 (6.3)
No Progression	5 (3.9)	
Progression- Required ITU Admission	3 (2.4)	
Total		127 (100)

## Discussion

The original Marshall score, previously known as the Multiple Organ Dysfunction score, was initially developed to help intensivists objectively measure the mortality risk of ICU patients from their clinical presentation encompassing six organ systems: cardiovascular, renal, respiratory, hepatic, haematological, and central nervous systems [7]. It was later then modified, simplified into three organ systems, and repurposed by the 2002 Revised Atlanta Classification as a risk stratification tool for acute pancreatitis and to recognise the need to properly escalate severe patients for organ support therapy [5]. Since then, amongst other risk stratification tools, the Modified Marshall score easily rose to attention and is widely recognised for its simplicity and easy applicability [7]. Our retrospective review, however, underscored the underutilisation of the Modified Marshall score amongst acute pancreatitis patients, with utilisation complexity and lack of awareness as the prominent barriers. 

**Figure 2:**
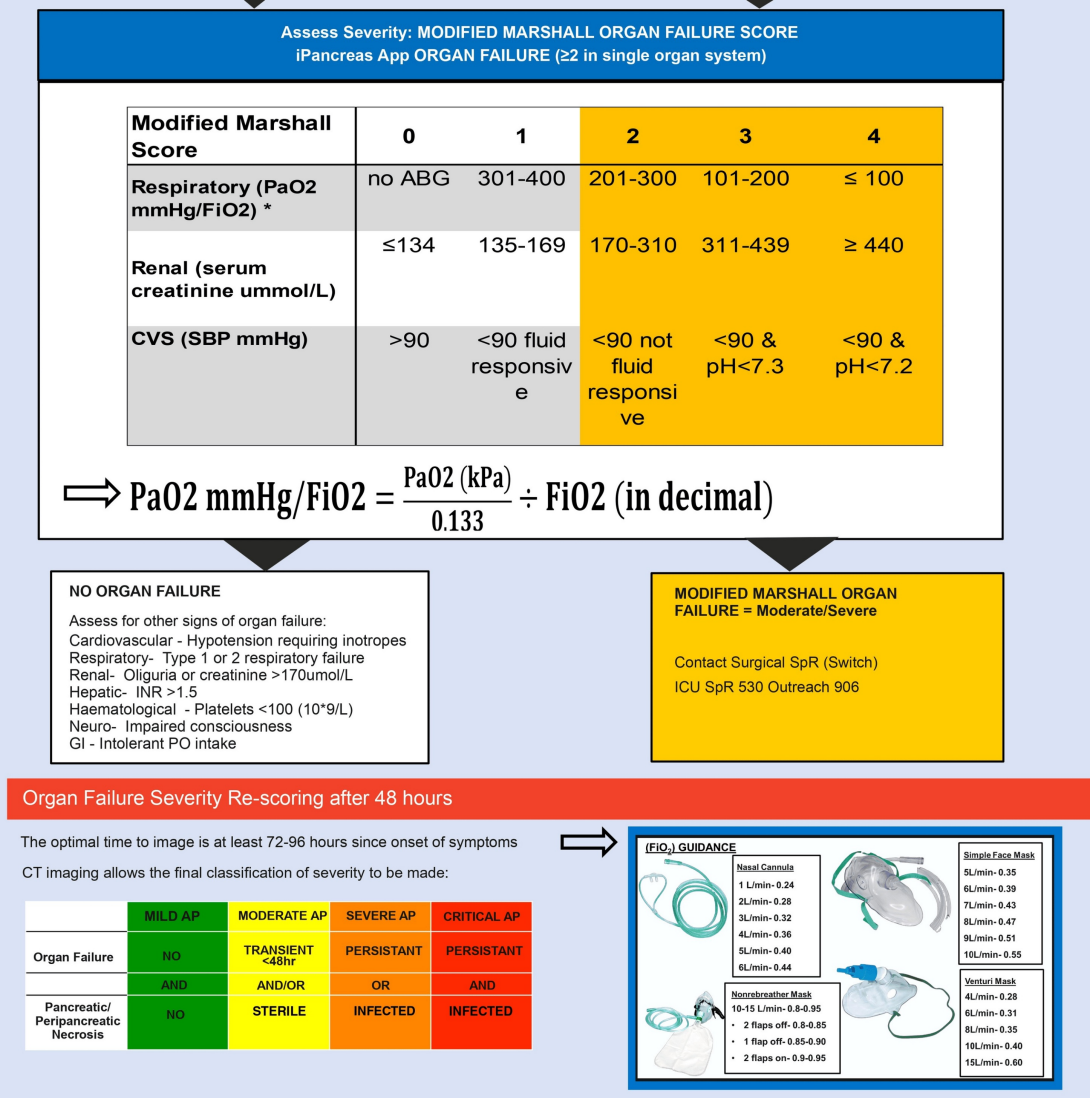
A segment of the newly redesigned poster for acute pancreatitis treatment pathway. The original version was developed and adapted locally by authors Salama & Young.  The first arrow elucidated the new simplified formula for the respiratory component (PaO2/FiO2) of the Modified Marshall Score. The second arrow showed the new FiO2 unit conversion guidance for different modes of oxygen delivery to aid the calculation of the MM score. This original framework is unpublished and owned by Young, who is also a co-author of the current project and thus provided as supplementary material. Permission for the use of the figure was obtained from the original authors.

**Figure 3:**
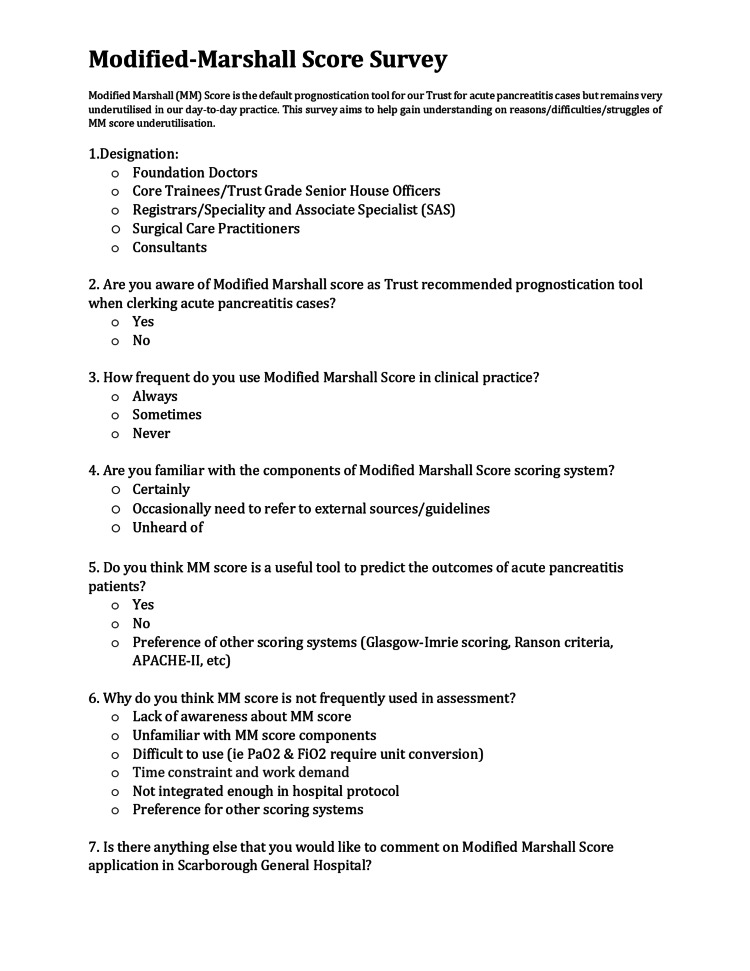
Survey questionnaire to explore reasons for underutilisation of Modified-Marshall Score.

**Figure 4:**
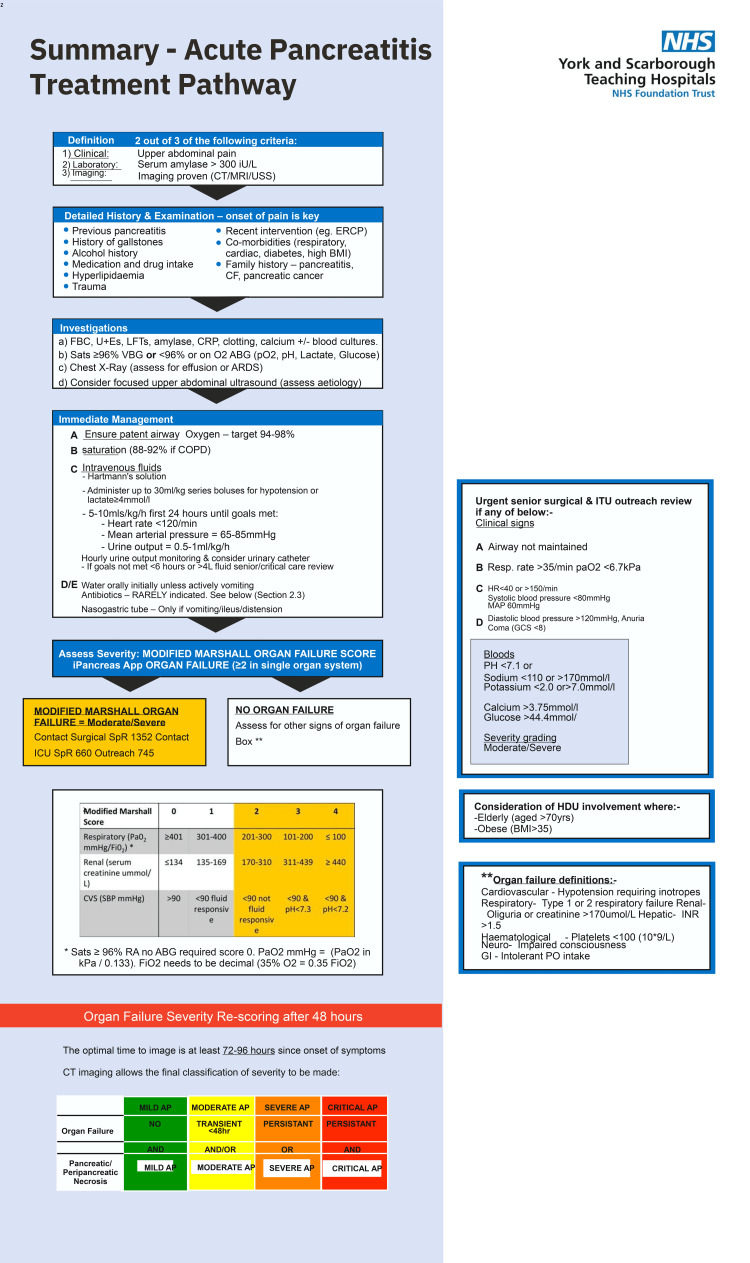
The original framework was designed and adapted locally by authors Young and Salama (2017), who present the Modified-Marshall scoring system in accordance with the Revised Atlanta Classification. This original framework is unpublished and owned by Young, who is also a co-author of the current project and thus provided as supplementary material. Permission for use of the figure was obtained from the original author. [5]

**Figure 5:**
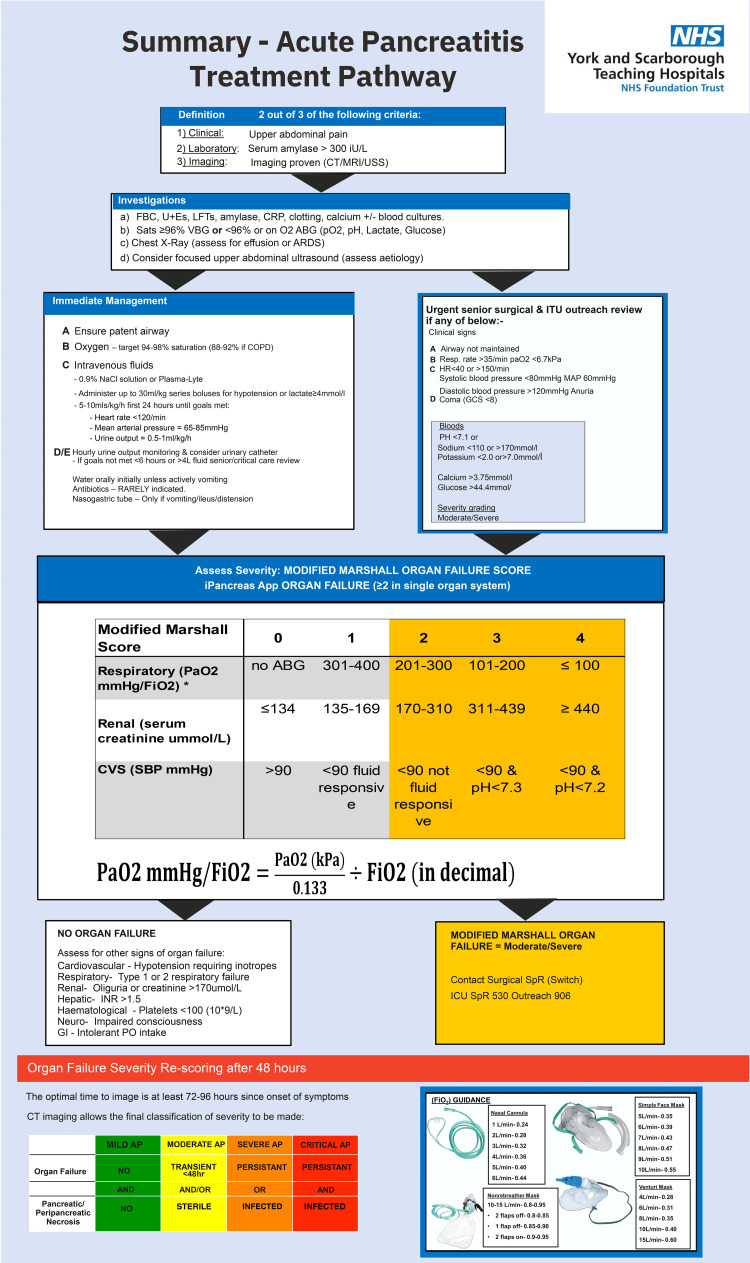
New framework designed following our retrospective review. Despite the simplified formula and unit conversion guidance included, the Modified-Marshall scoring system is in accordance with the Revised Atlanta Classification. Permission for use and adaptation of the figure was obtained from the original author, Young, who is also the co-author of this project. [5]

The respiratory component of the MM score (PaO2/FiO2), i.e., the ratio of arterial oxygen partial pressure (PaO2 in mmHg) to fractional inspired oxygen (FiO2 in decimal), is an evident challenge identified in our survey [10]. The calculation, per se, is not straightforward, as it requires manual conversion of the PaO2 value from kilopascals (kPa) to millimetres of mercury (mmHg) and the FiO2 value from oxygen flow rates in litres per minute (L/min) to decimal form (Figure 4 Appendix). To address this complexity, we redesigned the original poster to present a simplified version of the formula for the respiratory component (PaO2/FiO2) (Figure 2). In addition, we also developed new FiO₂ unit conversion guidance for different modes of oxygen delivery and requirements [10]. This guidance was placed alongside the formula for easier reference (Figure 2). The new changes were presented to all surgical clinicians in the departmental teaching, and a pocket edition of the new poster was distributed amongst all members of the department (Figure 5, Appendix). A review ensued over the subsequent one-month period to reassess the utilisation of the MM score following our interventions. Similarly, electronic records and surgical handover sheets were thoroughly assessed. We identified an improvement of 7/10 admissions with completed MM score risk stratification in a month. 

It is worth noting that there are many scoring systems established to help with clinical assessment of acute pancreatitis, such as HAPS (Harmless Acute Pancreatitis Score), JSS (Japanese Severity Score), BISAP (Bedside Index for Severity in Acute Pancreatitis), and APACHE II score [6,11-14]. It is also well-acknowledged that these systems are cumbersome or complex to use and that score-based evaluation cannot replace the assessment of an experienced clinician [6,15]. However, in our retrospective review, the Modified Marshall score demonstrated a relatively high negative predictive value (87.3%) (Table 2), and its utilisation complexity was evidently simplified. 

Interestingly, our data also suggested that the CT severity index tends to classify severe acute pancreatitis less frequently than other models compared to the Modified Marshall score and the Glasgow-Imrie score. This lower rate is likely due to the reason that radiographic evidence tends to lag behind clinical severity, leading to underestimation of disease severity by scoring systems that incorporate radiographic findings [15]. Organ failure secondary to systemic inflammatory response develops as early as 72 hours of presentation [4]. In contrast, radiological manifestations, including local and peripancreatic complications, often occur later in the disease course [1]. Peripancreatic fluid collection is classified as an early radiological manifestation (<4 weeks after onset of acute pancreatitis), while late radiological complications (>4 weeks after onset of AP) include pseudocysts and pancreatic or peripancreatic necrosis, whether sterile or infected [1]. 

The limitations of this study encompass its retrospective nature, potential for incomplete data, and potential for bias. Our sample size is small and may not be representative of the outcome of the entire practice. We also acknowledged that the short period of the second review can be a limitation, as further data would provide a more conclusive view on the utilisation of the Modified Marshall Score, following the new interventions applied.

It is also salient to highlight that our findings may be influenced by confounding factors such as pre-existing chronic organ dysfunction and varying numbers of active comorbidities, and that drawing definitive conclusions can be difficult. Acute pancreatitis may trigger pre-existing chronic organ dysfunctions such as chronic obstructive pulmonary disease (COPD), heart failure, and AKI, and increase the burden [2]. CKD patients with high but stable serum creatinine can cause misclassification of baseline severity with the MM score and will need manual monitoring of MM score progression for acute kidney injury [16]. Different aetiologies of acute pancreatitis were also included as a potential confounding factor, as different aetiologies are associated with varying disease severity and onset, which could independently influence MM scores and patient's actual outcome (Table 1) [2]. The small post-intervention review cohort and descriptive analysis limit inferential conclusions, but the trends support the feasibility and educational value of the intervention.

## Conclusions

Utilisation of the Modified Marshall score as a risk stratification tool for acute pancreatitis is still deficient in our local practice. Nevertheless, the MM score still demonstrated a high negative predictive value (NPV) in this cohort, as rarely do we see patients with a low MM score progress to requiring ITU interventions or emergency surgeries. Further validation in larger datasets would strengthen our findings. Our study both highlighted and addressed the need to simplify the utilisation complexity and reinforce awareness amongst surgical trainees.
